# Increased Cadmium Load, Vitamin D Deficiency, and Elevated FGF23 Levels as Pathophysiological Factors Potentially Linked to the Onset of Acute Lymphoblastic Leukemia: A Review

**DOI:** 10.3390/jpm14101036

**Published:** 2024-09-28

**Authors:** Vuk Djulejic, Ana Ivanovski, Ana Cirovic, Aleksandar Cirovic

**Affiliations:** 1 Faculty of Medicine, Institute of Anatomy, University of Belgrade, Dr Subotica 4/2, 11000 Belgrade, Serbia; vuk.djulejic@med.bg.ac.rs (V.D.); ana.zekavica@med.bg.ac.rs (A.C.); 2 Faculty of Medicine, University of Belgrade, Dr Subotica 4/2, 11000 Belgrade, Serbia; ivanovskia97@gmail.com

**Keywords:** acute lymphocytic leukemia, gut microbiota, Firmicutes, cadmium, vitamin D, anemia, iron deficiency anemia

## Abstract

The preventability of acute lymphocytic leukemia during childhood is currently receiving great attention, as it is one of the most common cancers in children. Among the known risk factors so far are those affecting the development of gut microbiota, such as a short duration or absence of breastfeeding, cesarean section, a diet lacking in short-chain fatty acids (SCFAs), the use of antibiotics, absence of infection during infancy, and lack of pets, among other factors. Namely, it has been shown that iron deficiency anemia (IDA) and lack of vitamin D may cause intestinal dysbiosis, while at the same time, both increase the risk of hematological malignancies. The presence of IDA and vitamin D deficiency have been shown to lead to a decreased proportion of Firmicutes in stool, which could, as a consequence, lead to a deficit of butyrate. Moreover, children with IDA have increased blood concentrations of cadmium, which induces systemic inflammation and is linked to the onset of an inflammatory microenvironment in the bone marrow. Finally, IDA and Cd exposure increase fibroblast growth factor 23 (FGF23) blood levels, which in turn suppresses vitamin D synthesis. A lack of vitamin D has been associated with a higher risk of ALL onset. In brief, as presented in this review, there are three independent ways in which IDA increases the risk of acute lymphocytic leukemia (ALL) appearance. These are: intestinal dysbiosis, disruption of vitamin D synthesis, and an increased Cd load, which has been linked to systemic inflammation. All of the aforementioned factors could generate the appearance of a second mutation, such as ETV6/RUNX1 (TEL-AML), leading to mutation homozygosity and the onset of disease. ALL has been observed in both IDA and thalassemia. However, as IDA is the most common type of anemia and the majority of published data pertains to it, we will focus on IDA in this review.

## 1. Introduction

Every third infant has iron deficiency anemia (IDA) [1]. IDA has been associated with the appearance of intestinal dysbiosis, and at the same time, an elevated risk of hematological malignancies [2]. On the other hand, every fifth cancer is related to intestinal dysbiosis [3], which means that intestinal dysbiosis is associated with an increased cancer risk. However, the clear relationship between these two entities (IDA and intestinal dysbiosis) and how IDA increases the risk of cancer is still unclear. It is crucial to thoroughly investigate and better understand the mechanisms involved in IDA-induced ALL occurrences.

The prevalence of vitamin D deficiency during childhood is also quite common, but varies depending on age and country, ranging from 12.1% [4] to potentially higher than 95% [5]. The link between iron deficiency and vitamin D deficiency is well established today [6]. Several studies have demonstrated a high prevalence of vitamin D deficiency among children with IDA [7,8]. Since vitamin D has potent anticancer effects, a deficiency in this vitamin is associated with an increased risk of malignancies [9].

Moreover, the link between pre-existing IDA and the subsequent appearance of acute lymphocytic leukemia (ALL) has not been thoroughly evaluated. The two-hit hypothesis predicts that the first appearance takes place in utero, while the second mutation responsible for the onset of the disease occurs in the post-natal period [10]. However, some circumstances regarding the second mutation are speculative. It is known to happen most likely due to the presence of chronic infection or inflammation [11]; nevertheless, some alterations in the function of the immune system are also required.

ALL affects slightly less than 5 out of 100,000 children [12]. The cumulative 5-year survival rate is slightly less than 90 percent, with pre-B ALL having a particularly favorable prognosis [13]. However, all survivors encounter a series of additional challenges, including compromised bone quality resulting from malignant cell infiltrations, as well as from the use of corticosteroids and chemotherapeutics [14]. Furthermore, the quality of life is diminished for all survivors of ALL [14]. Additionally, various social and socioeconomic challenges often arise in individuals after ALL [15]. There are a few known factors that influence the gut microbiome, such as the duration of breastfeeding [16], delivery method (vaginal or cesarean section), diet, and the use of antibiotics. Interestingly, exposure to pets during pregnancy and early childhood has been linked to a reduced risk of atopic and metabolic disorders due to changes in infants’ gut microbiota [17]. As our knowledge regarding the role of changes in gut microbiota in the pathogenesis of ALL in children grows, identifying factors that could influence gut microbiota becomes imperative. Therefore, in this review, we explored whether IDA, a common condition in infants and children, may contribute to the development of acute lymphoblastic leukemia (ALL).

Although an increased risk of hematological malignancies has been reported in both iron deficiency anemia (IDA) and thalassemia [2,18,19,20,21,22], IDA remains the most common type of anemia. As most of the published data focus on IDA, our review will primarily address this condition.

## 2. Adverse Effects of IDA and Vitamin D Deficiency on Gut Microflora: Reduced Percentage of Firmicutes in the Stool of Individuals with ALL

Ho and colleagues analyzed hematocrit levels and stool samples obtained from 80 preterm infants, revealing that the anemic group of infants had lower median percentages of Firmicutes. They also demonstrated a positive correlation between hematocrit levels and Firmicutes. This implies that the lower the hematocrit, the lower the proportion of Firmicutes [23]. Similarly, Díaz-Rodríguez analyzed stool samples from 18 children under 10 years of age. The study showed that children with IDA had a lower proportion of Firmicutes compared to children who recovered from IDA. Meanwhile, children without IDA had the greatest proportion of Firmicutes [24]. In vivo studies further confirmed these findings. Zhang et al. [25] treated Sprague Dawley rats with either a low-iron diet (12 μg Fe/g) or a regular diet (45 μg Fe/g). They found lower levels of acetic acid, butyric acid, and pentanoic acid in anemic rats with developed IDA compared to non-IDA rats [25]. Another in vivo study, performed by Sun et al., revealed that IDA mice had a decreased Firmicutes/Bacteroidetes ratio [26]. Xiao et al. reported significantly lower levels of butyrate in patients with inflammatory bowel disease who also had IDA compared to individuals with inflammatory bowel disease without IDA [27] Moreover, they found that butyrate can reduce the production of TNF-α and suppress inflammation [27]. Additionally, Soriano-Lerma et al. demonstrated the translocation of intestinal bacteria and increased blood levels of lipopolysaccharide in the serum of rats with IDA, which promotes the development of systemic inflammation [28].

Rajagopala et al. [29] analyzed stool samples obtained at the time of diagnosis of acute lymphocytic leukemia from 29 individuals aged 1–19 years and 25 healthy siblings. The study demonstrated that Firmicutes Faecalibacterium was significantly decreased at the time of diagnosis in ALL patients compared to control subjects [29]. Additionally, Bai et al. collected stool samples from 63 individuals, including 33 controls and 30 persons diagnosed with ALL, the majority of whom had B-cell acute lymphoblastic leukemia. They showed that bacteria under Firmicutes were significantly more abundant in the GI microbiota of controls compared to children with ALL [30]. Importantly, none of the included subjects had diarrhea in the month prior to stool collection and had similar dietary habits.

It has been shown that even low vitamin D supplementation (200 IU/d) can reverse changes in intestinal flora and optimize the immune response [31,32]. Interestingly, Singh et al. examined the association between gut microbiota and vitamin D plasma levels in 112 children aged 4–14 years from Qatar and found that children diagnosed with vitamin D deficiency had a lower abundance of Firmicutes, coupled with a higher Bacteroidetes to Firmicutes ratio [33]. Drall et al. [34] demonstrated that Firmicutes were significantly lower in Vitamin D-deficient children. A systematic review conducted by Bellerba et al. demonstrated that lower serum vitamin D concentrations are associated with a lower percentage of Firmicutes in the intestine [35].

Firmicutes are a significant source of short-chain fatty acids (SCFAs), such as acetate, propionate, and butyrate, in particular. The interaction between SCFAs and the immune system appears to be highly complex and not entirely understood. Recently, Liu and colleagues provided a comprehensive review of the effects of SCFAs on the immune system, noting that they affect almost every cell type involved in the immune system, including B-cells, T lymphocytes, neutrophils, macrophages, natural killer cells, eosinophils, and basophils [36]. SCFAs regulate host health by modulating both innate and adaptive immunity, with different SCFAs often having opposing effects on the same immune cell type [36]. Importantly, Yang et al. showed that SCFAs play a crucial role in promoting CD4+ lymphocytes to secrete IL-22, which protects intestines from inflammation, via the generation of hypoxia-inducible factor-1 (HIF-1) [37]. The deficiency of specific SCFA-producing taxa during the first year of life may prolong the maturation of gut microbiota towards the adult type.

Since the phylum Firmicutes produces butyrate, we can expect that a lower percentage of Firmicutes, often observed in children with IDA and ALL, would be associated with reduced circulating levels of butyrate. In addition to its beneficial effects on the intestinal mucosa, butyrate inhibits neutrophils from releasing pro-inflammatory cytokines (e.g., TNF-α and IL-17) and promotes the release of IL-10, which has anti-inflammatory effects [38].

**Figure 1:**
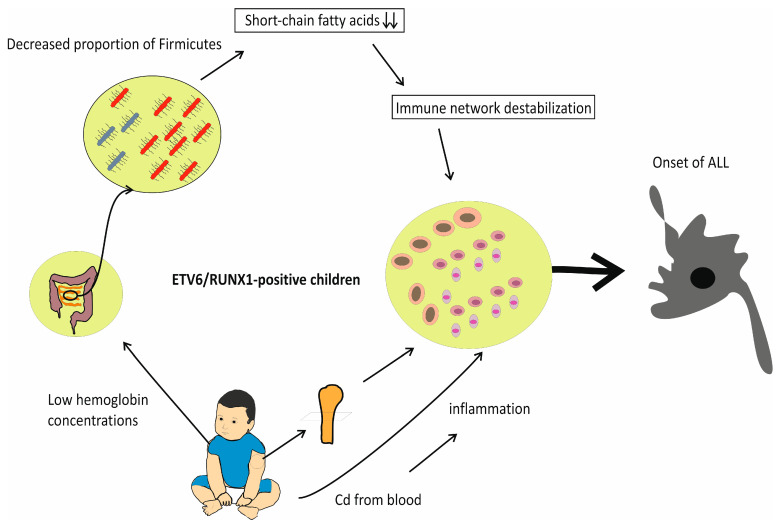
Mechanisms initiated by iron deficiency anemia which contribute to appearance of ALL.

This could lead to a lack of SCFA production due to a low percentage of Firmicutes, as speculated by Peppas and colleagues [11]. Moreover, they hypothesize that a deficiency in SCFAs could interfere with immune network stabilization, eventually resulting in chronic infection later in childhood, which could trigger the appearance of a second mutation [11] in children and the appearance of acute lymphoblastic leukemia which inherited the first mutation (e.g., ETV6/RUNX1) (Figure 1). In brief, we discussed that IDA may induce a deficiency of SCFAs by interfering with normal gut flora development.

## 3. Cd-Induced Toxicity in Bone Marrow

Furthermore, a chronically lowered iron concentration in the blood is associated with increased blood cadmium levels [39], a highly toxic heavy metal known for its pronounced pro-inflammatory characteristics [40]. As previously discussed, in subjects with iron deficiency, the duodenum exhibits an increased uptake of cadmium from food, facilitated by the divalent metal transporter-1 (DMT1). Notably, transferrin receptor 1 (TfR1) is highly expressed on the surface of bone marrow progenitors in cases of iron deficiency [41], particularly on specific progenitors like the common lymphoid progenitor [42]. While concentrations of heavy metals in bone marrow from individuals with leukemia have not been measured, it is established that blood levels of certain heavy metals, including cadmium, are elevated [43,44].

Speculatively, iron deficiency (ID) may promote cadmium bioaccumulation in the bone marrow since cadmium is transported through the blood bound to transferrin and subsequently deposits in tissues with a density of TfR1, which the bone marrow certainly possesses. The presence of cadmium in the bone marrow induces oxidative stress and inflammation which is evidenced by increased tissue concentrations of malondialdehyde (MDA) and reactive oxygen species (ROS) [45]. Suljevic et al. [46] exposed Japanese quails to cadmium via ad libitum water use for 20 days, and upon sacrifice, bone marrow obtained from the femur was analyzed. Their findings revealed a cadmium-induced decrease in cell surface area of pro-erythroblasts and lymphoblast type I, while lymphoblast type II was not detectable in the cadmium-exposed group [46], indicating a high severity of cadmium-induced toxicity in the bone marrow.

Cadmium activates the TNF-α/NF-κB pathway and induces inflammatory responses by increasing the expression of HO-1, IL-1β, iNOS, and COX2 [45,47]. Another study utilizing human tissue exposed to various doses of cadmium demonstrated that cadmium induces inflammation by increasing the expression of IL-1 [48]. Additionally, cadmium can interfere with proper DNA repair mechanisms [49]. On one side, cadmium accumulates in the bone marrow and induces oxidative stress and inflammation directly. However, it is also suggested that cadmium is capable of decreasing the proportion of Firmicutes in the intestine [47,50], and consequently, the concentrations of SCFAs drops as well. The NLRP3 inflammasome, responsible for the maturation and subsequent secretion of the inflammatory cytokines IL-1β and IL-18, is inhibited by the presence of SCFAs in the majority of tissues, except for the colon [51,52]. In contrast, Cd’s proinflammatory activity is mediated by activating the NLRP3 inflammasome [53]. Exposure to cadmium weakens the bond between enterocytes, making intestinal bacteria more likely to enter the bloodstream and potentially exacerbating systemic inflammation [45,50,54]. It is reasonable to speculate that a high Cd load, which happens in individuals with ID, may generate the perfect background for the genesis of the second mutation as it upregulates systemic and local inflammation. Moreover, Cd promotes a reduction in Firmicutes in the intestine and consequently reduces concentrations of SCFAs which may attenuate systemic inflammation.

Furthermore, Djulejic et al. recently discussed that insufficient blood concentrations of vitamin D may increase the risk of acute leukemia in at least two ways. The first suggests that cadmium blood concentrations could be elevated, particularly if calcium and iron blood levels are decreased, due to increased DMT-1 expression [55]; the second mechanism involves the lack of immunomodulatory effects produced by vitamin D insufficiency. Finally, Chrysochou et al. demonstrated that individuals diagnosed with ALL and AML (*n* = 133) had fourfold higher Cd blood concentrations compared to controls [44].

The interplay between IDA, vitamin D, cadmium, gut flora, and SCFAs levels and their impact on the immune system is highly complex. However, our simplified concept is clear and implies that IDA, vitamin D deficiency, and cadmium may lead to intestinal dysbiosis and a reduction in SCFAs. With this review, we have contributed a small piece towards answering one of the most significant and complex questions of the previous decades: the preventability of childhood ALL.

## 4. Maternal IDA during Pregnancy and Increased Risk of ALL in Children

One of the best examples reflecting the complexity of this issue is the finding that several studies have revealed a link between maternal IDA and an increased risk of ALL during childhood [56,57]. The in utero origins of childhood leukemia is currently receiving a lot of attention [58]. In a study from Taiwan, Orimoloye et al. examined the correlation between maternal IDA during pregnancy and the development of cancer in childhood. The study included over 2 million controls and more than 2000 children diagnosed with cancer. The results demonstrated that maternal IDA, when considering all types, was associated with a 90% higher risk for childhood leukemia, and specifically a 40% higher risk for ALL, compared to controls [56]. Another study from Denmark, which included over 6400 cases and 16,000 controls, found a 46% increased risk of ALL associated with maternal IDA during pregnancy [57]. It is reasonable to speculate that a similar pathophysiological mechanism could underlie the increased risk of ALL in childhood when considering IDA in both children and during pregnancy. Anemic women may have decreased vitamin D levels, though there are no data on whether maternal IDA could affect the gut microflora in newborns. As discussed earlier in our paper, Cd may play a role in the development of ALL in children, as cadmium primarily binds to transferrin, which is expressed in the placenta through transferrin receptor 1 [59]. Additionally, Cd may utilize various carriers, such as zinc transporters (e.g., ZIP8 and ZIP14) [60], iron transporter (DMT-1) [60], and calcium carrier (TRPV5 and TRPV6) [60], all of which are expressed in the placenta [59,61,62,63]. Consequently, Cd could potentially reach fetal bone marrow cells and accumulate there. The role of maternal Cd in the development of childhood ALL should not be dismissed and certainly warrants further investigation.

## 5. Fibroblast Growth Factor 23 (FGF23) in Acute Leukemia: The IDA-FGF23-Vitamin D Deficiency Axis

FGF23 is a protein synthesized by bone cells such as osteoblasts and osteocytes, whose main role is to reduce circulating levels of phosphate and vitamin D [64]. However, iron deficiency and cadmium exposure can increase blood levels of FGF23. For instance, Hanudel et al. found that wild-type C57BL/6 mice of both sexes, when fed a low-iron diet, had significantly higher levels of iFGF-23 compared to a group fed an adequate-iron diet [65,66]. The experiment lasted for eight weeks [65]. Another in vivo study demonstrated that wild-type mice exhibited elevated bone mRNA levels of iFGF23 [67]. A study involving 37 humans with autosomal dominant hypophosphatemic rickets (ADHR) and 158 healthy controls revealed that iFGF23 inversely correlated with serum iron levels [68]. Overall, iron deficiency may promote the production of iFGF23 [69] (iFGF23 is the active form of this 32 kDa glycoprotein, while cFGF23 is the inactive form). Additionally, Kido et al. found in their in vitro study that when UMR106 cells were exposed to cadmium (Cd), they had increased levels of iFGF23 in their supernatant [70]. There is also evidence that inflammation could upregulate FGF23 production, and inflammation is a key feature of Cd toxicity. Iron deficiency, Cd exposure, and inflammation could all promote the synthesis of iFGF23 and increase its plasma levels, which in turn interferes with vitamin D synthesis, leading to low circulating vitamin D levels [66]. Specifically, FGF23 reduces the activity of 25-hydroxyvitamin D-1α-hydroxylase [71]. Furthermore, increased plasma concentrations of FGF23 have been found in some hematological malignancies, such as acute leukemia [72] and myelodysplastic syndrome [73].

As FGF23 suppresses vitamin D synthesis, blood levels of vitamin D drop, and a deficiency in vitamin D is associated with an increased risk of acute leukemia [74,75,76]. A prospective study from northern India, which included 93 subjects aged 1 to 15 years diagnosed with ALL, found that almost 85% of them had vitamin D deficiency [74].

## 6. Discussion with Conclusions

**Figure 2:**
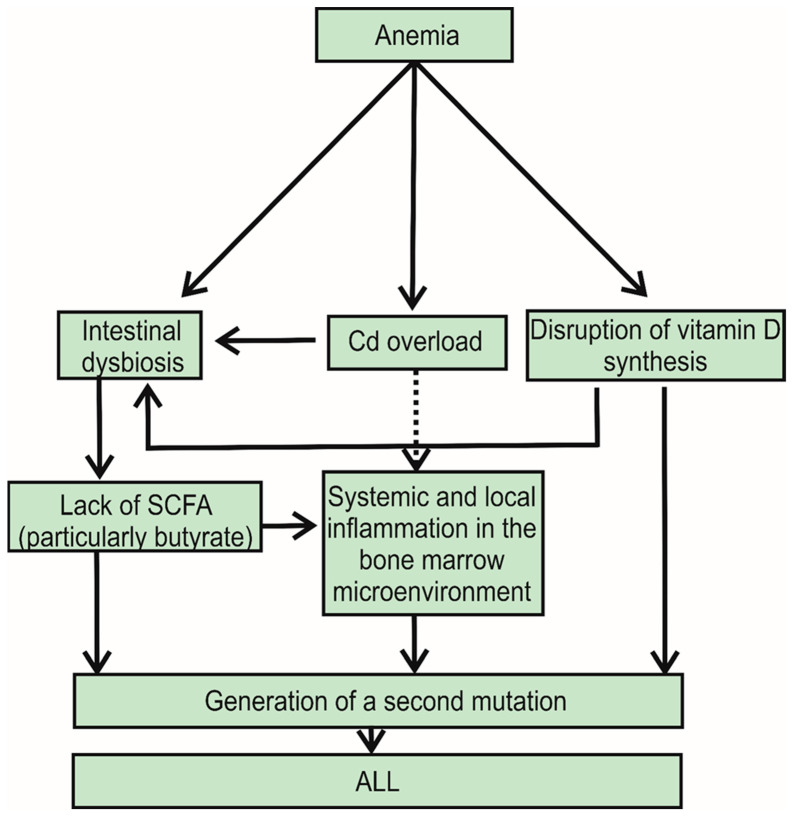
Summary of how iron deficiency anemia (IDA) can promote the onset of ALL in three different ways. The arrows used in this figure do not necessarily represent cause and effect but rather indicate associations between the connected boxes.

As a limitation of this review, we must mention that the pathogenesis of ALL is complex. ID and IDA, through mechanisms such as elevated FGF-23, lack of vitamin D, and increased cadmium load, may be considered contributing factors to the onset of acute lymphoblastic leukemia (Figure 2). For example, exposure to ionizing radiation increases the risk of leukemia onset independently of the mechanisms mentioned [77].

A question that naturally arises is whether all children are equally exposed to cadmium (Cd). As discussed, iron deficiency (ID), which is considered an intrinsic factor promoting increased Cd intestinal uptake and elevated Cd blood levels, is important. However, external exposure is also a significant issue [78]. External exposure includes factors such as living in polluted areas. For example, Stajnko et al. demonstrated that children living within 0.5–1 km of lead (Pb) smelters have higher Cd blood concentrations compared to children living in rural areas [79]. Children without ID or IDA living close to Pb smelters have 11% higher Cd blood levels [79]. García-Pérez et al. analyzed 638 cases of childhood leukemia and 13,188 children without hematological malignancies in Spain and reported an elevated risk of leukemia associated with living near industrial areas, particularly those where metals are processed [80,81]. Interestingly, Cd can downregulate the expression of several proteins involved in iron metabolism, such as intestinal heme carrier protein 1 and ferroportin 1, leading to decreased iron blood concentrations [82,83] which can ultimately result in ID and IDA. Cd also depletes hepatic and renal iron concentrations [83]. Thus, two possible scenarios emerge: first, ID causes increased Cd load, which in turn exacerbates ID; second, Cd exposure (e.g., from living near metal smelters) initiates ID, which then raises Cd blood concentrations. This creates a potential vicious cycle.

Therefore, it is essential to analyze all modifiable risk factors for iron deficiency (ID). Maternal IDA is associated with IDA in children, but beyond that, these factors can be broadly categorized as dietary and non-dietary [84]. Among dietary factors, exclusive breastfeeding for more than six months without introducing iron-rich foods can lead to the onset of IDA [85]. Additionally, preterm infants fed formula without iron supplementation are at a higher risk of developing IDA [86]. Regarding non-dietary factors, the presence of certain comorbidities, such as HIV and celiac disease [87,88], is strongly linked to the onset of IDA. Finally, prolonged consumption of large quantities of cow’s milk is one of the primary risk factors for the development of IDA. For instance, in toddlers, excessive milk intake is a common cause of IDA [89,90]. There are several mechanisms by which milk intake can induce ID. Calcium can reduce the rate of iron absorption; specifically, 1000 mg or more of calcium intake can decrease iron absorption by almost 50% [91]. Additionally, excessive milk ingestion can cause microscopic injuries and blood loss [92].

IDA may promote the development of ALL through at least three mechanisms. First, IDA can induce gut microbiota dysbiosis, which destabilizes the immune system. Second, IDA can potentiate cadmium Cd overload; Cd can induce intestinal dysbiosis and accumulate in bone marrow cells. Finally, IDA interferes with vitamin D synthesis, and vitamin D has anticancer effects. Additionally, individuals with IDA often have high levels of Cd, which induces systemic inflammation in the bone marrow microenvironment, creating conditions conducive to the genesis of a second mutation. Moreover, Cd itself promotes gut microbiota dysbiosis and reduces concentrations of short-chain fatty acids. Finally, iron deficiency and Cd exposure increase blood levels of FGF23, which in turn reduces blood concentrations of vitamin D, a deficiency of which is linked to the occurrence of ALL. Similar mechanisms could contribute to the appearance of childhood ALL in the case of maternal IDA in pregnancy.

By gaining a better understanding of how IDA promotes the onset of ALL, we could develop more effective preventive measures and reduce the incidence of ALL. IDA should not be overlooked, and its long-term adverse effects must be thoroughly understood.

IDA contributes to the development of acute lymphoblastic leukemia (ALL) through three main mechanisms: it causes intestinal dysbiosis, leads to cadmium (Cd) overload, and obstructs the synthesis of vitamin D. Both Cd and vitamin D deficiency further exacerbate intestinal dysbiosis. This dysbiosis results in a lack of short-chain fatty acids (SCFAs), particularly butyrate, which impairs butyrate’s positive effects on the immune system. Cd, on the other hand, induces inflammation. The combined effects of vitamin D deficiency, a destabilized immune system, and inflammation ultimately lead to the occurrence of a second mutation and the onset of ALL.

## Data Availability

Not applicable.
